# The safety and efficacy of miltefosine in the long-term treatment of post-kala-azar dermal leishmaniasis in South Asia – A review and meta-analysis

**DOI:** 10.1371/journal.pntd.0007173

**Published:** 2019-02-11

**Authors:** Joyce Pijpers, Margriet L. den Boer, Dirk R. Essink, Koert Ritmeijer

**Affiliations:** 1 Public Health Department, Médecins Sans Frontières, Amsterdam, The Netherlands; 2 Athena Institute, VU University Amsterdam, Amsterdam, The Netherlands; 3 Public Health Department, Médecins Sans Frontières, London, United Kingdom; Institute of Medical Sciences, INDIA

## Abstract

**Background:**

Miltefosine (MF) is the only oral drug available for treatment of visceral leishmaniasis (VL) and post-kala-azar dermal leishmaniasis (PKDL). Although the drug is effective and well tolerated in treatment of VL, the efficacy and safety of MF for longer treatment durations (>28 days) in PKDL remains unclear. This study provides an overview of the current knowledge about safety and efficacy of long treatment courses with MF in PKDL, as a strategy in the VL elimination in South Asia.

**Methodology/Principal findings:**

Literature was searched systematically for articles investigating MF treatment in PKDL. A meta-analysis included eight studies (total 324 PKDL patients) to estimate the efficacy of MF in longer treatment regimens (range 6–16 weeks). We found a per-protocol (PP) initial cure rate of 95.2% (95%CI 89.6–100.8) and a PP definite cure rate of 90% (95%CI 81.6–96.3). Descriptive analysis showed that 20% of patients experienced adverse events, which mostly had an onset in the first week of treatment and were likely to get more severe after four weeks of treatment. Gastrointestinal (GI) side effects such as vomiting, nausea, diarrhoea, and abdominal pain were most common.

**Conclusions/Significance:**

Longer treatment regimens with MF are effective in PKDL patients in India, however with the caveat that the efficacy has recently been observed to decline. GI side effects are frequent, although mostly mild or moderate. However, on the basis of limited data, we cannot conclude that longer MF treatment regimens are safe. Moreover, VL and PKDL pharmacovigilance studies indicate a risk for serious adverse events, questioning the safety of MF. The provision of safer treatment regimens for PKDL patients are therefore recommended. Until these regimens are identified, it should be considered to halt the use of MF monotherapy for PKDL in order to preserve the drug’s efficacy.

## Author summary

In this study, we reviewed the available literature on the subject of safety and efficacy of the oral drug miltefosine in the treatment of post-kala-azar dermal leishmaniasis (PKDL). Literature was searched systematically in the PubMed database and eight articles, with a total of 324 PKDL patients, were included. A meta-analysis was performed to estimate the percentage of patients cured after longer (>4 weeks) miltefosine treatment. An estimated 90% of patients was found to be cured one year after treatment with miltefosine. In addition, descriptive analysis showed that nearly 20% of the PKDL patients suffered from side-effects. The majority of these side-effects, such as vomiting, nausea, diarrhea and abdominal pain, were mild and related to the gastro-intestinal tract. The findings of this study show that miltefosine is effective, although the efficacy has been observed to decline. The gastro-intestinal side effects were frequent but mostly mild. However, based on the limited data in this study we cannot conclude that longer treatment regimens with miltefosine are safe. In order to preserve the drug’s efficacy, we suggest it may be put under consideration to halt the use of miltefosine monotherapy for PKDL until alternative treatment regiments (e.g. short combination therapies including miltefosine) are identified.

## Introduction

Post-Kala-Azar Dermal Leishmaniasis (PKDL) is a dermal complication of visceral leishmaniasis (VL) caused by the *Leishmania donovani* parasite, which is transmitted by *phlebotomine* sandflies. The PKDL disease can appear weeks to years after the successful cure of VL and is characterised by skin lesions, mainly present on places that are easily exposed to sunlight, such as the face [1]. The prevalence and severity of the disease vary between geographical regions. In East Africa, up to 60% of the former VL patients develop PKDL with mainly maculo-papular skin lesions, which are typically self-healing within three months. In South Asia, only 5–10% of the former VL patients develop PKDL. Most patients have hypopigmented macular lesions, however, up to 20% present with more severe papular or nodular skin lesions. Because spontaneous healing is probably limited [2,3], and may take years, treatment of more severe lesions is indicated. Considering PKDL cases are an important reservoir for transmission, potentially infecting new patients with VL [4], treatment is also required for public health reasons to achieve control of VL [1]. Because of the high endemicity limited to one geographical region and the availability of good diagnostic and treatment tools, in 2005 The Kala Azar Elimination Program was established as a regional initiative by the governments of Bangladesh, India and Nepal with the goal to eliminate VL in South Asia. Eliminating the PKDL reservoir is an important strategy in VL elimination.

The only oral drug available for the treatment of leishmaniasis is miltefosine (MF, hexadecylphosphocholine). This phospholipid derivative was originally developed as an anti-cancer drug but it was found to be unsafe after several studies indicated unacceptable renal- and gastrointestinal toxicity [5,6]. Scientists from Germany and the UK discovered the anti-leishmanial effect of the drug in the early 1990s. In 2003, MF was licensed for the treatment of VL [5]. The drug became the leading compound in the treatment of VL because it was effective, with limited side effects, and oral, so easy to administer [7]. In 2011, MF was added to the list of Essential Medicines by the WHO.

A substantial number of studies evaluated the safety and efficacy of MF in standard VL treatment of 28 days. Clinical trials have mainly been conducted in India, specifically in the state of Bihar, where VL is endemic [8]. Cure rates in VL patients range between 90–100% in a regular dose of 2.5 mg/kg per day for children aged 2–11 years; for people aged >12 years and < 25 kg body weight, 50 mg/day; 25–50 kg body weight, 100 mg/day; > 50 kg body weight, 150 mg/day; orally for 28 days. The safety concerns regarding MF mainly relate to its effect on the gastrointestinal tract [8]. Frequently observed adverse events in MF treatment regarding gastrointestinal toxicity that led to treatment interruption are nausea, vomiting, loss of appetite and diarrhoea. Other commonly observed toxicities are related to liver- and renal functions (e.g. elevated creatinine and ALT and AST levels). However, these are often not clinically relevant and normally stabilize during treatment [8]. In addition, animal studies have showed teratogenicity and impaired fertility in men and women, meaning that the use of MF could negatively influence the fetal congenital development. Impaired male fertility in humans as a consequence of miltefosine is currently under assessment by the FDA.

Miltefosine was first used in treatment of PKDL in 2006 [9]. In comparison to VL, PKDL requires longer treatment durations with MF. The drug is currently used as first-line treatment for at least twelve weeks in PKDL infected patients in India, Nepal, and Bangladesh [10]. PKDL requires longer treatment durations because of the limited skin penetration of antileishmanial drugs, and the fact that there is no other clinical marker for cure than disappearance of lesions, which may take more than one year in case of macular lesions [1]. Only few studies have investigated the safety and efficacy of the long-term MF treatment for PKDL and sample sizes in those studies are relatively small. Due to the slow clearance of MF in the body concerns are raised regarding the safety and efficacy of the drug in long-term treatment for PKDL. Therefore, this study aims to provide an overview of the current knowledge about safety and efficacy of longer treatment regimens (>28 days) with MF in PKDL patients, in order to contribute to the control of leishmaniasis.

## Methods

### Study design

This was a systematic review including a quantitative meta-analysis of data from different studies, in order to provide more accurate estimates of the effects of MF treatment in PKDL patients. This study was carried out in accordance with the Preferred Reporting Items for Systematic Reviews and Meta-Analyses (PRISMA) statement [11].

### Search strategy

**Fig 1:**
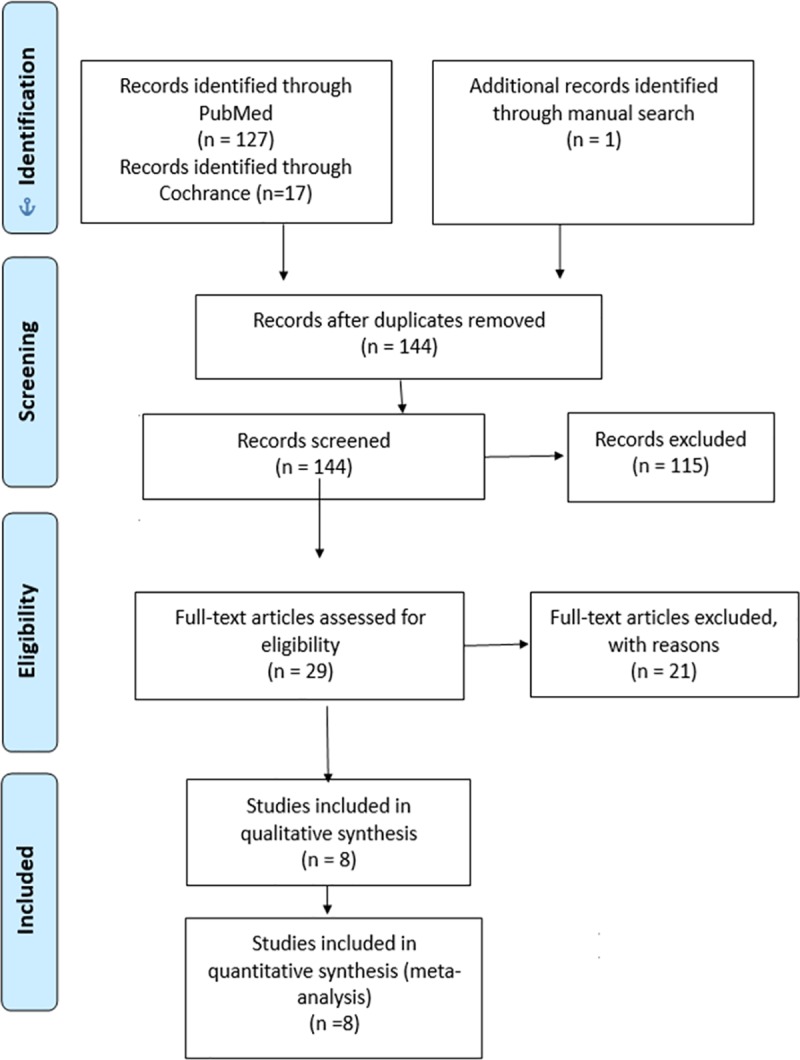
PRISMA flow diagram.

The databases PubMed and Cochrane library were searched systematically using the following search terms: *Miltefosine or hexadecylphosphocholine*, *Post-kala-azar dermal leishmaniasis*, *visceral leishmaniasis*, *kala-azar*, *safety*, *efficacy*, *tolerability*, *toxicity*, *clinical effectiveness*, *adverse events and South-Asia*, *India*, *Nepal*, *Bangladesh* (Table 1). The total number of hits was 146. Fig 1 shows the flow diagram of the literature search. In addition to the computer search, reference search of all reviewed articles was performed to identify articles missed through the database search. One article was found manually.

**Table 1:** Search strategies for databases PubMed and Cochrane.

Search terms	Hits	Included
**PubMed**		
(Miltefosine OR Hexadecylphosphocholine) AND (PKDL OR Post-Kala-Azar Dermal Leishmaniasis OR VL OR Visceral Leishmaniasis OR Kala-Azar OR Back Fever) AND (Efficacy OR Clinical effectiveness OR Safety OR toxicity OR Tolerability OR adverse events) AND (South Asia OR South-East Asia OR India OR Bangladesh OR Nepal)	129	7
**Cochrane**		
(Miltefosine OR Hexadecylphosphocholine) and (Efficacy OR Safety OR Toxicity OR adverse events) and (Visceral Leishmaniasis OR Kala-azar OR PKDL OR ''Post-kala-azar dermal leishmaniasis'')	17	1 (duplicate)

Inclusion criteria were miltefosine monotherapy, VL or PKDL, human study population, and articles had to be written in English. Articles were excluded based on geographical location (America, Europe and Africa were excluded), in case the study used MF for treatment courses of 28 days or less and in case the study was conducted on animals. There were no further restrictions on age, sex or publication date.

### Data extraction

All included articles were assessed on basic characteristics such as aim, methodological approach, sample size, treatment dose, treatment duration, conclusions and scientific quality. Primary outcomes of the current review were efficacy and safety. Efficacy was expressed in per-protocol (PP) cure rates and Intention-to-treat (ITT) cure rates at the end of treatment (i.e. initial cure rate) and at the end of follow up (i.e. definite cure rate).

Safety was displayed in adverse events and abnormal haematological parameters during or after treatment with MF. The seriousness of these toxicities was rated according to the Common Terminology Criteria for Adverse Events (CTC) of the National Cancer Institute [12]. Grades ranged from 1 to 5 (mild, moderate, severe, life-threatening and death). In case of mild and moderate severity (CTC grade 1 and 2, respectively), patients had to be treated with additional medication. In case of severe and life-threatening severity (CTC grade 3 and 4, respectively), treatment with MF had to be discontinued.

### Statistical analysis

Data management and analyses were performed using SPSS version 25.0 [13]. Pooled estimates of initial and definite PP cure rates were calculated by random-effects regression analysis, using Wilson’s Macros for meta-analysis (Wilson, version 2005.05.23), after applying sample weights according to sample size. Moderator (subgroup) analysis was performed to indicate estimated cure rates for different duration treatment groups (a dummy variable was created for 6, 8, 12 and 16 weeks of treatment). Heterogeneity between studies was assessed using Cochran’s Q included in the meta-analysis function. A p-value of <0.05 indicated significant heterogeneity.

## Results

Eight experimental articles were included for analysis in the current study [14–21]. Table 2 provides an overview of the characteristics and main findings regarding efficacy and safety of MF in the included studies. All studies investigated longer treatment regimens of >28 days with MF in PKDL patients with the WHO-recommended standard dosing of (approximately) 2.5mg/kg/day, and were all originated from India. A total number of 324 patients were treated with MF, divided over a total of eleven treatment arms. Treatment duration ranged from six to sixteen weeks. One study investigated patients treated with MF for six weeks [21], in four study arms patients were treated with MF for eight weeks [15,18–20], in five study arms patients were treated for 12 weeks [14–18], and in one study patients were treated for 16 weeks [21].

**Table 2:** Characteristics and safety and efficacy data of the included studies.

*Study*, *year*	*Design*	*N total (treatment arm)*	*Treatment dose and duration*	*Efficacy* *Initial cure rate* *(95%CI)*	*Efficacy* Definite cure rate (95%CI) [ITT%]**	*Safety*
Moulik et al [14]	Randomized controlled trial	184 (1)84, (2)98	(1) MF 100mg/day for 12 weeks (2) LAmB* 5mg/kg body weight i.v. twice weekly for 3 weeks	N.A.	(1) 100% *[45]*	N.A.
Ramesh et al [15]	Clinical trial, Cohort, Prospective, 18 months follow up	86 (1)60, (2)26	(1) MF 100 mg/day for 12 weeks (2) MF 150 mg/day for 8 weeks	(1) 100% (2) 76.5%	(1) 89.5% (78.9–95.1) *[75]* (2) 68.8% (44.4–85.8) *[43]*	Anorexia CTC 1 (n = 1) Vomiting CTC 2 (n = 7) Elevated SGOT and SGPT CTC 2 (n = 3)
Sundar et al [16]	Exploratory clinical trial	33 (1)28 (2)5	(1), Patients ≥ 25kg) MF 100 mg/day for 12 weeks (2, Patients < 25kg) MF 50 mg/day for 12 weeks	N.A.	(1+2) 96.6% *[85]*	Vomiting and diarrhoea CTC 4 (n = 1)
Ghosh et al [17]	Single arm open label trial	27	(Patients ≥ 25kg) MF 100 mg/day for 16 weeks (Patients < 25kg) MF 50 mg/day for 16 weeks (Patens age 2–11) MF 2,5mg/kg/day for 16 weeks	N.A.	12 weeks: 57% 16 weeks: 100% *[55]*	Severe abdominal pain CTC 2 (n = 6) Nausea and vomiting CTC 2 (n = 3) Nausea, vomiting and abdominal pain CTC 3 (n = 2) CVA*** CTC 4 (n = 1)
Sundar et al [18]	Open-label, randomised, parallel-group multicentric trial	36 (1)18, (2)18	(1) MF 100 mg/day (patients ≥ 25 kg) or 50 mg/day (patients <25kg) for 8 weeks (2) MF 100 mg/day (patients ≥ 25 kg) or 50 mg/day (patients <25kg) for 12 weeks	(1) 100% (2) 94%	(1) 81% (57–93) *[76]* (2) 93% (71–95) *[78]*	Diarrhoea CTC 1 (n = 1) Vomiting CTC 1 and 2 (n = 8) Elevated bilirubin CTC 2 (n = 1)
Ramesh et al [19]	Open, single-arm study	26	MF 150 mg/day for 60 days	96% (79–99)	100% *[92]*	Severe abdominal pain CTC 3 (n = 1) Diarrhoea CTC 1 (n = 2) Vomiting CTC 1 (n = 7)
Modak et al [20]	Clinical trial, single arm	6	MF 100mg/day for 8 weeks	100%	100% *[100]*	Diarrhoea CTC 1 (n = 1) Nausea CTC 1 (n = 2) Vomiting CTC 1 (n = 1) Abdominal pain CTC 1 (n = 1)
Jha et al [21]	Escalating-dose, open-label, phase 2 trial	120 (1)30, (2)30, (3)30, (4)30	**(1)** MF 50 mg/day for 6 weeks (2) MF 50 mg/day for 1 week + 100 mg/day for 3 weeks (3) MF 100 mg/day for 4 weeks (4) MF 100 mgs/day for 1 week + 150 mg/day for 3 weeks	100%	(1) 93% (78–99) *[100]*	Frequent GI toxicity: vomiting and diarrhoea in 62% of patients Elevated serum asparate aminotransferase CTC 2 (n = 1)

* Liposomal Amphotericin B

** Intention-To-Treat cure rate

*** CVA: Cerebrovascular Accident

There was some variation in methodological approaches between the included studies. First, all studies had an experimental design of which three were randomized controlled trials [14,18,21]. Furthermore, three of the included studies were single-arm trials [17,19,21], and five studies had two or more study arms [14–16,18,21]. Of those five studies, one study compared MF with another treatment (i.e. liposomal amphotericin B) [14] and the remaining four studies investigated different MF treatment durations [15,16,18,21]. Secondly, patients were treated as outpatients in five studies [14,15,17–19] while the rest of the included studies treated patients as inpatients (i.e. in hospitals) [16,20,21]. Thirdly, cure rates were assessed in two different ways. Three studies used parasite load measures by quantitative PCR (qPCR) at the end of treatment and at the end of follow up to indicate cure [14,15,17]. The remaining five studies assessed cure rate based on clinical features at the end of treatment and at the end of follow up [16, 18–21]. In those studies, patients were labelled cured if lesions had disappeared after treatment with MF. There was some variation in the length of follow up period between the studies. Two studies used a follow up period of six months [14,21], five studies used a follow up period of twelve months [15,16,18–20] and in one study a follow up period of eighteen months was used [17].

### Efficacy

**Fig 2:**
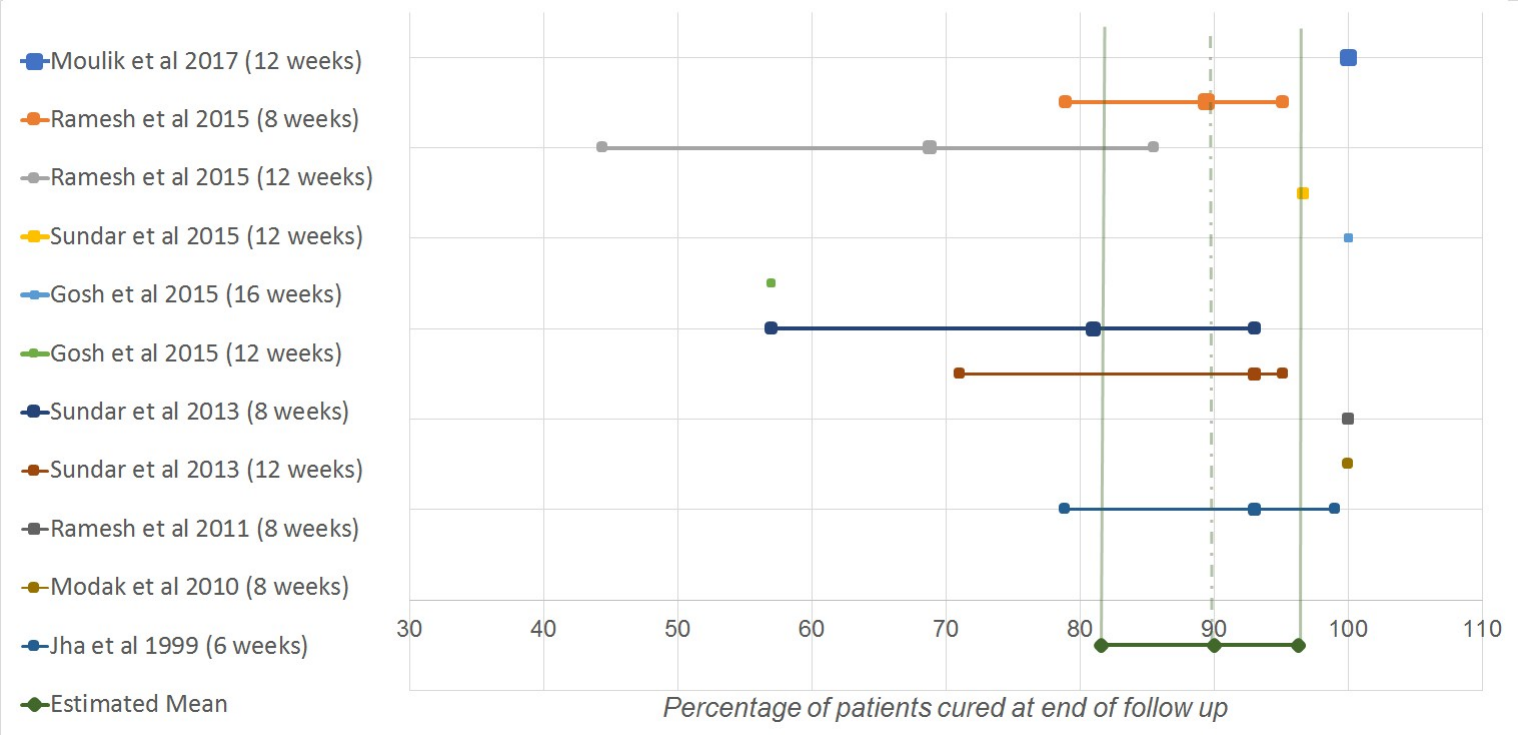
Forest plot definite cure rates. Studies are arranged by publication year. A larger sized square indicates greater weight.

The cure rates at the end of follow up (definite cure) per study and the results of the meta-analysis are displayed in Fig 2. Meta-analysis showed an estimated PP definite cure rate of 90.0% (95%CI 81.6–96.3) and the average ITT cure rate was 74.9%. The lowest PP and ITT definite cure rates, 57% and 55%, respectively, were found in the study of Ghosh et al [17]. These numbers are substantially lower than the definite cure rates found in the other studies, which can be explained by the high number of treatment discontinuations due to severe side-effects in this study. In several study-arms, all patients, in at least one trial arm, were cured at 12-month follow up [14,17,19,20]. As can be seen in Table 2, the ITT definite cure rates ranged from 43–100%. The low ITT cure rates in the studies of Moulik et al [14], Ramesh et al [15] and Ghosh et al [17] (45%, 43% and 55%, respectively), are strongly influenced by high lost-to-follow-up in those studies. In the study of Moulik and colleagues [14], the drop-out-rate was no less than 57%. In the 8-week study arm of Ramesh et al [15] and in the study of Ghosh et al [17] the drop-out-rates were 33% and 35%, respectively.

**Fig 3:**
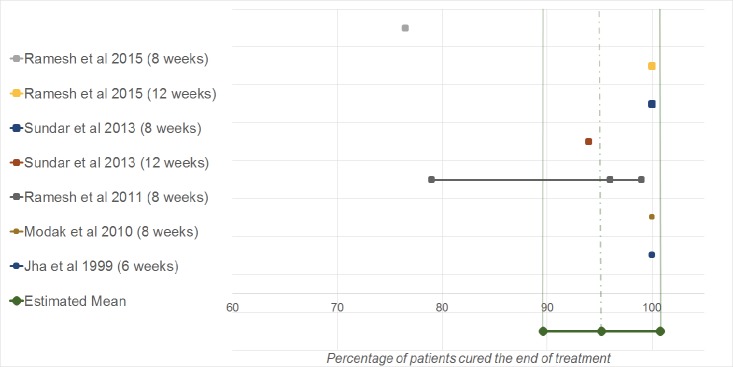
Forest plot initial cure rates. Studies are arranged by publication year. A larger sized square indicates greater weight.

The cure rates at the end of treatment (initial cure) per study and the results of the meta-analysis are presented in Fig 3. Five of the eight included studies reported an initial cure rate [15,18–21]. Meta-analysis showed an estimated per protocol initial cure rate of 95.2 (95%CI 89.6–100.8).

As can be seen in Figs 2 and 3, there seem to be outliers regarding both the initial and definite cure rates (i.e. numbers that lay outside of the 95%CI of the pooled estimates), which indicates heterogeneity. Analysis indicated the degree of variance in and between studies. In the analysis for initial cure rate, significant heterogeneity was indicated (Q = 15.6, I^2^ = 61.6% and P<0,05). 61.6% of the variance can be contributed to true heterogeneity. In the analysis for definite cure rate, no significant heterogeneity was indicated (Q = 13.4, I^2^ = 25.1 and P>0,05). 25.1% of the variance can be contributed to true heterogeneity.

### Subgroup analysis

In addition to the estimated overall cure rates, subgroup meta-analysis was performed to indicate the estimated cure rates per treatment group related to treatment duration. Table 3 shows the outcomes of this analysis with treatment duration as moderator variable. No significant differences were found in initial and definite cure rates between the different treatment durations.

**Table 3:** Meta-analysis. Estimated cure rates with treatment of MF for different treatment durations.

	Treatment duration (k)	Estimated effect	SE	95% CI
**Initial cure rate**	6-week treatment (1)	100*	7.36	85.5–114.4
	8-week treatment (4)	93.1	3.68	85.9–100.3
	12-week treatment (2)	97.0	5.32	86.5–107.4
**Definite cure rate**	6-week treatment (1)	93*	12.44	68–117
	8-week treatment (4)	92.6	6.22	80.4–104.8
	12-week treatment (5)	83.1	5.57	72.2–94.0
	16-week treatment (1)	100*	12.44	75.6–124.4

(k)number of treatment arms for which cure rates were provided in the corresponding article

*Only one study (treatment arm) investigated this treatment duration which resulted in a broader confidence interval and a greater standard error.

Studies that were conducted in the past five years show a lower average cure rate (92.6% and 85.7% for initial and definite cure, respectively) than studies that were conducted more than five years ago (98.7% and 97.7% for initial and definite cure, respectively). However, these differences were not statistically significant (*p* = 0.142 and *p* = 0.081 for initial and definite cure, respectively).

### Safety

Nearly 20% (n = 64) of all patients experienced adverse events. The most common side effects reported in the included studies are related to gastrointestinal (GI) adverse events. GI side-effects reported were nausea, vomiting, abdominal pain, diarrhoea or a combination of these events. All included studies reported that vomiting occurred in the majority of their patients. Vomiting was graded CTC 1 or 2 in nearly 10% of all patients (n = 20), however data was lacking in most studies regarding those mild and moderate adverse events. Vomiting with CTC grade 3–4 was experienced by three patients. In addition to vomiting, abdominal pain was reported in three studies (n = 10 patients) and graded CTC 1–3. In patients that experienced events graded CTC 3 or 4, treatment was discontinued. Events graded CTC 1 or 2 were treated symptomatically. In one study, six patients were treated with additional medication (Ondansetron) prior to taking MF in order to reduce repeated vomiting (CTC grade 2) [15]. In one study [17], treatment was reduced to twelve weeks because of unacceptable side effects.

Besides observable side effects, six studies provided data on haematological and laboratory tests performed before, during and after treatment. Laboratory abnormalities were seen in liver function (elevated bilirubin, SGOT and SGPT) and kidney function (elevated creatinine and serum asparate aminotransferase). However, in all but one patient, all of these laboratory abnormalities were non-severe and stabilised during treatment without interventions (e.g. additional medication, or treatment interruption). In one patient, an elevated bilirubin sample was graded CTC 2 [18].

In addition to the above-mentioned adverse events, one patient suffered from a cerebrovascular accident (CVA) [17]. This serious neurological condition (CTC grade 4) had most likely occurred as a result of the treatment with MF [17]. Ghosh et al [17] investigated the causality association between MF and the CVA with the ‘Naranjo adverse drug reaction probability scale’ [17]. However, an explanation for this association was not provided in the article.

The data provided about the time of onset of MF side-effects was lacking in the included articles. The studies of Ramesh et al [15] and Sundar et al [16] did not mention at what time during or after treatment the reported adverse events had occurred. In two studies was mentioned that the GI side-effects occurred during the first weeks of treatment. A few studies provided more concrete data on the days, or weeks, of onset of adverse events. In one study, unacceptable GI side-effects started after four weeks of treatment [15]. In addition, one study provided information on the day of onset for all gastrointestinal side effects [18]. The days of onset for vomiting graded CTC1 were: 32, 33, 38, 39, 48, 52 and 69, and vomiting graded CTC2 were: 33 and 77. Overall, adverse events were likely to occur in the first week of treatment, but became more severe after six weeks.

## Discussion

This study aimed to review the efficacy and safety of longer MF treatment regimens in PKDL patients. Meta-analysis showed an estimated cure rate of 95.2% and 90% for PP initial and definite cure rates, respectively. The average ITT cure rate was 74.9%. These findings are similar to literature investigating the efficacy of MF in treatment of VL with a duration of 28 days or less. Dorlo et al [8] found definite cure rates for VL ranging from 80–100% in their review. Furthermore, 97.3% of the 1100 VL patients in a large phase IV trial were cured after 28-day treatment with MF (93.2% by ITT analysis) [22]. In addition, 95% of these patients were cured at 12-month follow up (82% by ITT analysis) [22]. With regard to different treatment durations, subgroup analysis in this review showed no significant difference in initial or definite cure rates. However, the sample size of this study was small, and therefore identifying the most effective duration of MF treatment in PKDL patients requires further research.

Concerns were raised about potential toxicities as a result of the slow clearance of MF in the body, drug accumulation, and the lack of studies investigating long-term treatment. This review found that severe GI side-effects such as vomiting, nausea, abdominal pain and diarrhoea were experienced by nearly 20% of the PKDL patients. Dorlo et al [8] found similar side-effects in their review of 28-day treatment with MF for VL and explain that the GI side-effects can be attributed to MF’s working on the mucosa of the gastrointestinal tract. The current review found that adverse events in PKDL patients became more severe later in treatment (i.e. after six weeks). This can be explained by the long half-life of MF (approximately seven days) and the increasing drug levels in the patients over time. Contrary, in the trial of Bhattacharya et al [22], VL patients experienced more adverse events in the first week of treatment and those events diminished towards the end of the 28 days treatment. Bhattacharya et al [22] explained that the decrease of events over time might be a result of the rapid resolution of the VL disease features.

In the current review, one patient experienced a CVA (CTC4), which was assessed to be related to MF. To our best knowledge, this has not been seen in previous MF toxicity studies. There are, however, other severe incidental side-effects reported in VL studies, that were most likely related to MF treatment. In a VL study in India, a twelve-year-old boy was diagnosed with Steven-Johnson Syndrome (CTC4) [23]. Furthermore, one study reported the case of a male VL patient that developed fatal acute pancreatitis (CTC5) on the 13^th^ day of treatment with MF [24]. Two recent studies conducted in Bangladesh [25,26] described five cases of ophthalmic issues (annular corneal ulcer, Mooren’s ulcer, and marginal keratitis) as a complication of the 12 weeks MF regimen in PKDL patients. In four cases the problems were reversible after discontinuation of MF. In the fifth case, MF treatment was continued as the issues were not reported. As a result, the patient has now permanent disability and blindness in the affected eye [25,26].

Phase I and II trials in the field of cancer research have indicated frequent toxicities and a lack of therapeutic efficacy in cancer patients treated with MF [27–32]. Similar to the findings in this review, the majority of side-effects were gastrointestinal. In the study by Berdel et al [28,29], 70% of the lung cancer patients treated with MF for nine weeks experienced episodes of nausea and vomiting. In the study of Unger et al [30], nearly 90% of the breast cancer patients experienced gastrointestinal side effects when treated with 100–150 mg MF daily for nine weeks. Similar results were found in a phase II trial where 90% of the cancer patients experienced episodes of nausea and vomiting when treated with MF for six weeks [31]. In addition to the gastrointestinal issues, another study indicated renal toxicities in 30% of their patients during MF treatment with doses up to 200mg per day (median treatment duration was six weeks) [32].

A challenge with MF is the reproductive toxicity. Embryo-fetal toxicity, including death and teratogenicity, was observed in embryo-fetal studies in rats and rabbits administered oral miltefosine during organogenesis at doses that were respectively 0.06 and 0.2 times the maximum recommended human dose (MRHD), based on body surface area (BSA) comparison. Numerous visceral and skeletal fetal malformations were observed in a fertility study in female rats administered miltefosine prior to mating through day 7 of pregnancy at doses 0.3 times the MRHD [33]. Therefore, female PDKL patients of child-bearing age are required to take contraceptives during and for five months after treatment with MF in order to prevent potential fetal congenital abnormalities. In addition to the teratogenicity, reduced fertility is seen in male VL patients treated with MF. Van Thiel et al [34] showed that 62% (n = 21) of the male military patients diagnosed with cutaneous leishmaniasis (CL) and treated with 150mg MF for 28 days experienced reduced ejaculation volume.

Despite the convenience of an oral treatment, patients are likely to poorly adhere to a twelve-week treatment that involves taking medication two times a day, when given non-directly observed. Because PKDL patients are typically not sick, the experience of frequent GI side-effects due to MF can easily result in missed doses and/or early discontinuation of treatment [35]. The reviewed articles showed relatively high dropout rates in groups with longer treatment durations, as a result of GI-side effects. For this reason, it was suggested that MF should be administered under clinical observation [6]. However, the practical feasibility of directly observed treatment administration can be questioned.

With regard to the non-adherence to MF treatment, Dorlo et al [8] emphasize the issue of loss of drug sensitivity and resistance that could lead to a decrease in the life-span of MF. While Dorlo et al [8] describe the drug non-susceptibility *in vitro*, while it is not yet demonstrated *in vivo*, more recent (case) studies indicate the increasing drug unresponsiveness and relapse rate in both VL and PKDL patients after MF monotherapy [8, 36–39]. The availability of expensive MF in the private sector in India ten years ago contributed to the persistence of sub-therapeutic dosage, resulting in drug-unresponsiveness [8,35]. In order to respond to the risk of resistance, the use of short combination therapies with MF is recommended. As an oral compound, MF has great potential to be used in multiple drug therapy for short durations (10 to 14 days). However, pharmacokinetic data show that it takes at least two weeks before MF reaches therapeutic blood levels [8]. Further research is necessary to identify safe and effective short combination therapies including MF in the treatment of PKDL patients.

### Strengths and limitations

The strength of this study is the meta-analytic design. Literature on the safety and efficacy of long-term treatment with MF is scarce and sample sizes are small. Therefore, combining the existing studies in a meta-analysis provides a more accurate estimate of the cure rates in endemic populations in South Asia. However, the results of this review need to be seen in the light of some limitations. First, all included studies were conducted in India, mainly in the state of Bihar. Although the majority of patients treated with long-term MF are Indian patients, one should be careful to generalise the results of this study to other endemic countries in South Asia. Secondly, the meta-analysis showed significant heterogeneity between studies, indicating that the variation in and between the studies was not based on standard error alone but can be contributed to methodological variations between studies (e.g. different assessments of cure, inpatient versus outpatient, and different research designs). Thirdly, the results of later studies may be affected by a decreased susceptibility to miltefosine and the overall efficacy we found may no longer reflect the reality on the ground.

### Conclusion

In order to eliminate kala-azar in South Asia, PKDL patients need to be treated effectively. This review showed that treatment regimens with MF of six weeks or longer are effective (up to 90%) in PKDL patients in India, however with the caveat that the efficacy has recently been observed to decline. There is no straightforward answer to whether MF is an appropriate choice for the treatment of PKDL. This review showed that GI side effects are frequent in longer MF treatments, although mostly limited to mild or moderate side effects. However, on the basis of limited data included in this review, we cannot conclude that longer MF treatment regimens are safe. Moreover, information from previous VL studies and PKDL pharmacovigilance indicate a risk for serious, irreversible or even fatale adverse events, questioning the safety of longer treatment regimens with MF.

The highly common GI side effects can lead to non-compliance and form a risk for drug resistance. For this reason, directly observed treatment where possible, adequate surveillance of MF susceptibility in both PKDL and VL patients, as well as drug sensitivity monitoring in parasite isolates is required.

The provision of other treatment regimen for PKDL patients are highly recommended. It may be put under consideration to halt the use of miltefosine monotherapy for PKDL and proceed with safer alternative regimen. This will also help preserve the drug’s efficacy. In parallel, research into new treatment regimens should be encouraged.

## Supporting information

S1 File Meta-analysis database. (XLSX)

S2 File PRISMA checklist. (DOC)
